# Long-term survival benefit of ruxolitinib in a patient with relapsed refractory chronic active Epstein–Barr virus

**DOI:** 10.1007/s00277-019-03647-5

**Published:** 2019-03-04

**Authors:** Zhili Jin, Yini Wang, Jingshi Wang, Jia Zhang, Lin Wu, Zhao Wang

**Affiliations:** Department of Hematology, Beijing Friendship Hospital, Capital Medical University, 95 Yong An Road, Xicheng District, Beijing, 100050 China

Dear Editor,

Chronic active Epstein–Barr virus (CAEBV) often succumbs to hemophagocytic lymphohistiocytosis, multi-organ failure, or Epstein–Barr virus–positive lymphomas if untreated. The only proven effective treatment for the disease is hematopoietic stem cell transplantation (HSCT), although the incidence of transplantation-related complications is high. New alternative treatment should be sought for CAEBV patients who are not eligible for, or refuse to do, transplantation. We report a case of long-term asymptomatic survival of a CAEBV patient under ruxolitinib treatment.

A 9-year-old male patient was admitted to the local hospital due to “intermittent fever” on September 29, 2017. The highest temperature recorded was 39.8 °C. The result of EBV-DNA was 8.5E+03 copies/ml. Enhanced abdominal CT showed splenomegaly. The child still had recurrent fever under treatment of ganciclovir. During October 17 to 20, 2017, body temperature was reduced to normal with methylprednisolone treatment (40 mg). During October 21 to 29, 2017, the patient was treated with methylprednisolone (30 mg) and fever recurred during the period. PET/CT showed symmetric enlargement of the cervical lymphadenopathy and hepatosplenomegaly. A left cervical lymph node needle biopsy was done. Pathological diagnosis was EBV-positive lymphoproliferative disease, grade I–II, with focal necrosis. On December 1, 2017, 7.5 mg of dexamethasone was given orally as well as etoposide was given. The patient still had recurrent fever. A treatment plan of allo-HSCT was recommended. However, the family members considered the high risk and refused transplantation. On December 10, 2017, EBV-DNA (8.5E+05 copies/ml) was detected, ruxolitinib (5 mg bid) was given orally, and the patient’s temperature returned to normal in 1 day. EBV-DNA (2.5E+05 copies/ml) was detected in March 2018, and ruxolitinib was reduced to 2.5 mg bid orally; EBV-DNA (whole blood) (1.3E+05 copies/ml) and EBV-DNA (plasma) (< 5E+02 copies/ml) were detected in June 2018 and ruxolitinib was reduced to 1.25 mg bid orally. EBV-DNA (whole blood) (4.4E+03 copies/ml) and EBV-DNA (plasma) (< 5E+02 copies/ml) were detected in August 2018, sCD25 was1071 pg/mL (normal range < 6400 pg/mL), NK cell activity 12.81% (normal range > 15.11%), and a CD107a test showed a normal result. Abdominal ultrasound showed normal spleen size. The patient was maintained at a dosage of 1.25 mg bid of oral ruxolitinib and follow-up was conducted in January 2019. Since beginning ruxolitinib treatment, the patient’s body temperature has remained normal and the spleen has gradually shrunk to a normal size range.

CAEBV is refractory to antiviral therapy, intravenous immunoglobulin, interferon, and conventional chemotherapy and many other treatments have been tried including corticosteroids or cyclosporine, autologous EBV–specific cytotoxic T cells, and thus has a poor prognosis [1]. Ruxolitinib can inhibit JAK activity by competitively inhibiting the ATP binding site of JAK kinase, may decrease the viable cell number of EBV-positive NK or T cell lines and PBMCs from patients with CAEBV, suppress the production of inflammatory cytokines of CAEBV patients, and suppress the phosphorylation of STAT3 in EBV-positive NK or T cell lines [2]. In our research, since beginning ruxolitinib treatment, the patient’s body temperature has remained normal, the spleen has gradually shrunk to a normal size range and the copies of EBV-DNA is decreased which is similar to the research above mentioned. However, some studies have shown that ruxolitinib may have, in conjunction with other immunosuppressive factors such as asplenia and long-term steroid treatment, led to EBV reactivation [3]. Therefore, the relationship between EBV and Ruxolitinib still needs more attention and more cases to be verified.

## Abbreviations

CAEBV: Chronic active Epstein–Barr virus
PBMCs: Peripheral blood mononuclear cells
HSCT: Hematopoietic stem cell transplantation
JAK: Janus kinase
STAT: Signal transducer and activator of transcription proteins
